# Prevalence and risk factors of active pulmonary tuberculosis among elderly people in China: a population based cross-sectional study

**DOI:** 10.1186/s40249-019-0515-y

**Published:** 2019-01-18

**Authors:** Can-You Zhang, Fei Zhao, Yin-Yin Xia, Yan-Ling Yu, Xin Shen, Wei Lu, Xiao-Meng Wang, Jin Xing, Jian-Jun Ye, Jian-Wei Li, Fei-Ying Liu, Jian-Lin Wu, Lin Xu, Hui Zhang, Jun Cheng, Li-Xia Wang

**Affiliations:** 1 National Center for Tuberculosis Control and Prevention, Chinese Center for Disease Control and Prevention, Beijing, China; 2 Heilongjiang Provincial Center for Tuberculosis Control and Prevention, Harbin, Heilongjiang China; 3 Shanghai Municipal Center for Disease Control and Prevention, Shanghai, China; 4 Jiangsu Provincial Center for Disease Control and Prevention, Nanjing, Jiangsu China; 5 Zhejiang Provincial Center for Disease Control and Prevention, Hangzhou, Zhejiang China; 6 Henan Provincial Center for Disease Control and Prevention, Zhengzhou, Henan China; 7 Hubei Provincial Center for Disease Control and Prevention, Wuhan, Hubei China; 8 Center for Tuberculosis Control of Guangdong Province, Guangzhou, Guangdong China; 9 Guangxi Provincial Center for Disease Control and Prevention, Nanning, Guangxi China; 10 Sichuan Provincial Center for Disease Control and Prevention, Chengdu, Sichuan China; 11 Yunnan Provincial Center for Disease Control and Prevention, Kunming, Yunnan China

**Keywords:** Prevalence, Risk factor, Pulmonary tuberculosis, Aged/elderly

## Abstract

**Background:**

The problem of population aging is a critical public health concern in modern China, and more tuberculosis (TB) control efforts are needed to reach elderly people at high priority. In this study, we aim to determine the prevalence and identify the risk factors of TB among elderly people in China.

**Methods:**

A multistage cluster-sampled cross-sectional survey was conducted in 2013, and 27 clusters were selected from 10 counties of 10 provinces in China. All consenting participants greater than or equal to 65 years of age were screened for pulmonary TB with a chest X-ray (CXR) and a symptom questionnaire. Three sputum specimens for bacteriological examination by microscopy and culture were collected from those whose screening was positive. Prevalence was calculated, a multiple logistic regression model was performed to confirm the risk factors, and population attributable fraction (PAF) of each risk factor was calculated to indicate the public health significance.

**Results:**

Of 38 888 eligible people from 27 clusters, 34 269 participants finished both questionnaire and physical examination. There were 193 active pulmonary TB cases, 62 of which were bacteriologically confirmed. The estimated prevalence of active pulmonary TB and bacteriologically confirmed TB in those 65 years of age and older was 563.19 per 100 000 (95% *CI*: 483.73–642.65) and 180.92 per 100 000 (95% *CI*: 135.89–225.96), respectively. Male sex, older age, living in rural areas, underweight, diabetes, close contact of pulmonary TB (PTB) and previous TB history are all risk factors for TB. The risk of TB increased with increasing age and decreasing body mass index (BMI) after adjusting for other factors, and there is a positive dose–response relationship.

**Conclusions:**

In China, active case finding (ACF) could be implemented among elderly people aged 65 and above with underweight, diabetes, close contact history and previous TB history as a priority, which will get significant yields and be cost-effective.

**Electronic supplementary material:**

The online version of this article (10.1186/s40249-019-0515-y) contains supplementary material, which is available to authorized users.

## Multilingual abstracts

Please see Additional file 1 for translations of the abstract into the five official working languages of the United Nations.

## Background

Population aging is a critical public health concern in modern China. The proportion of elderly people aged 65 and above was 10.8%, and people aged 60 and above 16.7% of a population of 1382 million people in 2016 [1]. The risk of tuberculosis (TB) increases with age, as shown by studies internationally [2–5] and in China [6]. Almost half (48.8%) of the diagnosed TB cases in China’s Fifth National Prevalence Survey of TB were elderly people, among whom 39.8% were asymptomatic, and 53.2% didn’t seek any medical care [7]. Bele et al. found that population aging was one of the bottlenecks in TB control in rural China, and more TB control efforts are needed to reach the most vulnerable populations at high priority [8].

Rapid case identification and early treatment are the most important interventions to prevent TB transmission and reduce its incidence [9, 10]. Systematic screening of high-risk groups to diagnose TB early has proven effective to help end the global TB epidemic [11, 12]. However, it’s not feasible to screen all elderly people due to the huge population numbers in China. On the other hand, combining age with one or more TB risk factors could detect a higher TB prevalence in a more concentrated population. In this study, we aim to determine the prevalence and identify the risk factors of TB among elderly people to shape the TB screening algorithm for this high-risk population in China.

## Methods

### Study setting and sampling

This was a cross-sectional study. Sample size was estimated using a method appropriate to estimate a single population proportion. The 369/100000 prevalence of bacteriologically positive pulmonary TB (PTB) among elderly people (≥ 65 years) from the most recent national TB prevalence survey was used as the estimated prevalence of elderly population (unpublished data). We assumed 95% confidence interval and 0.2 allowable error and used the formula $n=\frac{pq}{{\left(\frac{d}{{\mathrm{Z}}_{\upalpha}}\right)}^2}$ (*p* = 369/100000, q = 1 – p, d = 0.25p, α = 0.05, *Z*_α_ = 1.96). A design effect of 1.8 from the simple random sampling was considered. So, a total of 29 873 elderly participants were needed and a sample size of 33 192 was determined to allow 10% of non-response.

We applied the multistage sampling in 27 study sites (10 townships in rural areas and 17 communities in urban areas) from ten counties of ten provinces in China. In the first stage, ten out of 31 provinces were selected, of which three were from eastern China, three from central China, three from western China, and one from four municipalities directly under the central government (Beijing, Shanghai, Tianjin and Chongqing). Selection at this stage was also based on willingness to participate and the human resources of each province. In the end, Sichuan, Guangxi, Yunnan of western China, Heilongjiang, Henan, Hubei of central China, Zhejiang, Jiangsu, Guangdong of eastern China, and Shanghai were chosen. In the second stage, one county per district that had more than 500 000 people was randomly selected in each province. In the third stage, random cluster sampling was used to select communities or townships. If the total number of general population in selected community or township was less than 30 000, then the nearest community or township would also be included in the study site, to reach 30 000. Finally, a total of 38 888 elderly people were eligible participants in the study.

### Definitions

Suspected TB symptoms were defined by this study, as any of the following conditions: (1) cough for more than 2 weeks; (2) hemoptysis; (3) cough for more than 1 week yet less than 2 weeks, and accompanied with any of the following symptoms: fever, chest pain, night sweating, loss of appetite, fatigue, and/or weight loss (> 3 kg). The definitions of previous TB cases, human immunodeficiency virus or acquired immune deficiency syndrome (HIV/AIDS), known diabetes, close contacts, underweight, tobacco use, drinking history, chronic bronchitis, average family annual income per capita and average family living area, are shown in Table 1. It is necessary to note that China has its own criteria of body mass index (BMI) for adults: underweight BMI < 18.5, normal 18.5 ≤ BMI < 23.9, overweight BMI ≥ 24 [13].

**Table 1:** Definitions of terms used in this study

Term	Definition
Previous TB cases	Registered in TB Management Information System, and finished treatment or cured.
HIV/AIDS	Registered in local CDC database, whom were diagnosed according to diagnostic criteria for HIV/AIDS published by National Health Commission of the People’s Republic of China in 2008.
Known Diabetes	Recorded on the Citizen Health Management Files as diagnosed with Diabetes (fasting plasma glucose level ≥ 7.0 mmol/L, or 2-h plasma glucose level ≥ 11.1 mmol/L), plus those using medicine to control blood glucose by self-report.
Close Contacts	Living with new active PTB case for at least 7 days in the 3 months before diagnosis.
Underweight	BMI < 18.5, i.e. Weight (kg)/Height × 2 (m) < 18.5.
Tobacco use	Ever smoked tobacco by self-report.
Drinking history	Drinking more than one unit (21 g pure alcohol) per week by self-report.
Chronic bronchitis	Chronic bronchitis history by self-report.
Average family annual income per capita	Average annual income per capita of urban family = RMB 27000 (USD 3970), average annual income per capita of rural family = RMB 8000 (USD 1176), (USD 1 = RMB 6.8).
Average family living area per capita	Average of urban family = 29 m^2^, average of rural family = 31 m^2^

### Data collection

In our study, each province organized a research team of 50–100 staff for data collection, including researchers, health-care workers, enumerators, and local government staff. From June to September 2013, participants were interviewed for any suspected TB symptoms. Meanwhile, information of participants’ sex, age, marital status, education, medical history, smoking and drinking habit, and socioeconomic status were collected. Their height and weight were measured to calculate the BMI as an indicator of nutritional status. All participants were offered chest X-ray (CXR) examination. The interviews took approximately 15–20 min each CXR was completed in less than 1 h including waiting time for one participant.

Participants with any one of the suspected TB symptoms or CXR abnormalities consistent with TB were asked to submit three sputum samples (morning, night and spot sputum) for sputum smear and culture. Patients with smear-positive and/or culture-positive sputum were diagnosed as bacteriologically positive TB. Patients with active PTB included those with bacteriologically positive sputum, and those diagnosed only by changes on their chest radiographs—known as clinically diagnosed PTB [6].

**Fig. 1:**
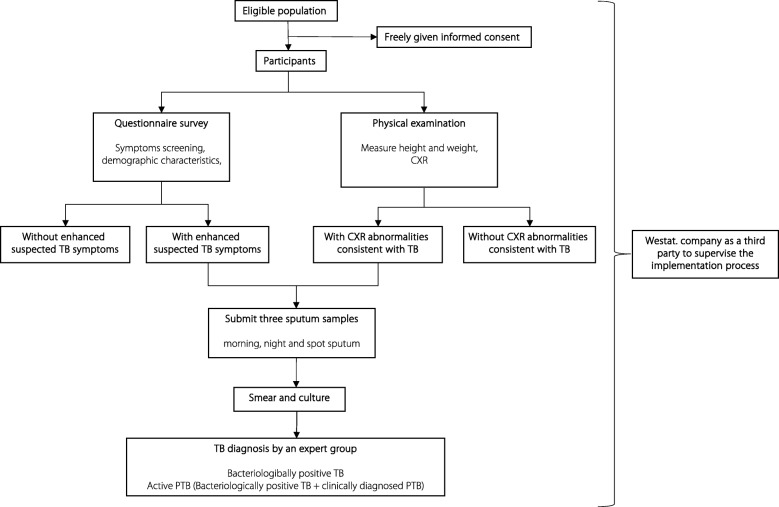
Flowchart of the survey process in China in 2013. CXR: Chest X-ray; TB: Tuberculosis; PTB: Pulmonary tuberculosis

Each county in China, according to the national guideline [14], has a TB diagnosis group, composed of at least three health staff, including a clinical doctor, a laboratory technician and a radiologist. They are trained to diagnose active PTB cases based on patient symptoms and clinical history, radiographic findings, bacteriological results, and response to antibiotics. A national expert group reviewed the data from each patient involved in this study to confirm the diagnosis. Quality checks were done according to the national guidelines [14]. The survey process is shown in Fig. 1.

The data collected was reviewed by the enumerators carefully on the same day, and any mistakes were corrected within 24 h. During the investigation period, 5 % of questionnaires were randomly selected for review by supervisors. Meanwhile, the study invited the Westat Company (Rockville, Maryland, USA) as a third party to supervise the implementation process.

### Statistical analysis

All data were double entered using an online input system developed by a local software company. TB prevalence of subgroups was respectively calculated, including bacteriologically positive PTB and active PTB. Chi-square (*χ*^2^) test or Fisher’s exact test was used to compare the difference of subgroups. Variables identified as potentially relevant for active PTB included: sex, age, residence, marital status, education level, family average annual income per capita, family living area per capita, tobacco use, drinking history, diabetes, close contacts, previous TB cases, and chronic bronchitis. Univariate logistic regression analysis was used to identify potential risk factors associated with active PTB. To control potential confounders, a multiple logistic regression model with stepwise selection was performed. All statistical tests were two-tailed, and the significance level was set at *P* = 0.05 or less. Odds ratio (*OR*) with 95%confidence interval (*CI*) was calculated for categorical variables in the study to assess the strength of association between risk factors and TB prevalence. All tests were performed using SAS 9.3 (SAS Institute Inc., Cary, North Carolina, USA).

The population attributable fraction (PAF) is most commonly defined as the proportional reduction in average disease risk over a specified time interval that would be achieved by eliminating the exposure(s) of interest from the population while distributions of other risk factors in the population remain unchanged [15, 16]. Formula as below was used to calculate PAF of each adjusted risk factor.

$$ PAF=\frac{p_e\left( RR-1\right)}{p_e\left( RR-1\right)+1} $$

*p*_*e*_ = proportion of source population exposed to the factor of interest. RR (risk ratio) may be the ratio of two cumulative incidence proportions, two (average) incidence rates (rate ratio), or an approximation of one of these ratios. In this study, *p*_*e*_ means proportion of population exposed to adjusted risk factors, and *OR* was used to replace RR.

### Ethical considerations

The study was reviewed and approved by the Institutional Review Board of Chinese Center for Disease Control and Prevention. Written informed consent was signed by each participant before enrollment. All patients identified were referred to the local designated TB clinic or hospital for treatment according to national guidelines [14].

## Results

### Demographic characteristics

There were 38 888 eligible elderly people (≥ 65), of which 34 269 (88.12%) participants were included in this study and finished both the questionnaire and physical examination. Of these 18 212 were male (53.14%) and 16 057 were female (46.86%) (Table 2). The median age was 72 (interquartile range: 68–77).

**Table 2:** Demographic characteristics of the elderly participants aged 65 or over in China in 2013

	Number	Proportion (%)
All	34 269	100.00
Sex		
Female	18 212	53.14
Male	16 057	46.86
Age group		
65–74	21 685	63.28
75–84	10 658	31.10
≥ 85	1926	5.62
Residence		
Urban	12 933	37.74
Rural	21 336	62.26
Marital status		
Married	24 953	72.82
Single/divorced	8091	23.61
Unknown	1225	3.57
Education level		
Unknown	1249	3.64
Illiterate or semi-illiterate^a^	12 092	35.29
Elementary school	12 581	36.71
Secondary school	7123	20.79
College and above	1224	3.57
Family annual income per capita		
Higher than average	10 854	31.67
Lower than average	23 415	68.33
Family living area per capita		
Higher than average	16 434	47.96
Lower than average	17 835	52.04

^a^semi-illiterate: people who did not complete elementary school

### Prevalence of bacteriologically positive TB and active PTB

Among the 34 269 participants, 817 (2.38%) reported to have one or more of the TB symptoms identified, 2542 (7.42%) had CXR abnormalities, and 168 (0.49%) had both.

A total of 62 bacteriologically positive TB cases and 193 active PTB cases were identified. The prevalence of bacteriologically positive PTB and active PTB were 180.92/100000, and 563.19/100000 respectively. The prevalence of bacteriologically positive TB and active PTB were higher in males than in females, increased with age and were higher in rural over urban areas (Table 3).

**Table 3:** TB Prevalence and comparison of subgroups of the elderly participants aged 65 or over in China in 2013

Category	Number of participants	Bacteriologically confirmed TB cases					Active TB cases				
		Number	Prevalence (95% *CI*)	*χ* ^2^	*P* value	Crude *OR* (95% *CI*)	Number	Prevalence (95% *CI*)	*χ* ^2^	*P* value	Crude *OR* (95% *CI*)
All	34 269	62	180.92 (135.89–225.96)				193	563.19 (483.73–642.65)			
Sex											
Female	18 212	17	93.35 (48.97–137.72)	16.51	< 0.0001	ref	49	269.05 (193.72–344.39)	60.05	< 0.0001	ref
Male	16 057	45	280.25 (198.37–362.14)			3.00 (1.72–5.26)	144	896.81 (750.33–1043.28)			3.35 (2.42–4.64)
Age(years)											
65–74	21 685	22	101.45 (59.06–143.85)	25.76	< 0.0001	ref	93	428.87 (341.70–516.03)	22.58	< 0.0001	ref
75–84	10 658	30	281.48 (180.75–382.20)			2.70 (1.56–4.69)	79	741.23 (577.77–904.68)			1.72 (1.27–2.33)
≥ 85	1926	10	519.21 (197.40–841.02)			3.93 (1.81–8.55)	21	1090.34 (624.00–1556.69)			2.12 (1.30–3.44)
Residence											
Urban	12 933	14	108.25 (51.55–164.96)	6.07	0.0137	ref	35	270.63 (180.97–360.28)	31.75	< 0.0001	ref
Rural	21 336	48	224.97 (161.33–288.62)			2.08 (1.15–3.78)	158	740.53 (625.06–856.00)			2.75 (1.91–3.97)
Marital status											
Married	24 953	43	172.32 (120.82–223.83)	0.51	0.7769	ref	137	549.03 (457.09–640.97)	0.78	0.6777	ref
Single/divorced	8091	17	210.11 (110.23–309.99)			1.21 (0.69–2.12)	47	580.89 (414.82–746.97)			1.05 (0.75–1.46)
Unknown	1225	2	163.27 (19.76–589.39)			0.73 (0.18–3.03)	9	734.69 (336.33–1394.29)			1.03 (0.53–2.03)
Education level											
Unknown	1249	2	160.13 (19.38–578.06)	5.89	0.2076	–	9	720.58 (329.86–1367.49)	9.64	0.0469	6.41 (0.81–50.58)
Illiterate or semi-illiterate	12 092	28	231.56 (145.79–317.33)			–	76	628.51 (487.21–769.82)			6.84 (0.95–49.16)
Elementary school	12 581	24	190.76 (114.44–267.09)			–	77	612.03 (475.33–748.74)			6.99 (0.97–50.21)
Secondary school	7123	8	112.31 (48.43–221.26)			–	30	421.17 (270.46–571.88)			4.84 (0.66–35.48)
College and above	1224	0	0 (0–301.47)			ref	1	81.7 (2.07–455.07)			ref
Family annul income per capita											
Higher than average	10 854	10	92.13 (35.03–149.24)	6.93	0.0085	ref	44	405.38 (285.60–525.16)	7.50	0.0062	ref
Lower than average	23 415	52	222.08 (161.72–282.44)			2.41 (1.23–4.75)	149	636.34 (534.17–738.52)			1.57 (1.12–2.20)
Family living area per capita											
Higher than average	16 434	26	158.21 (97.40–219.02)	0.90	0.3422	ref	77	468.54 (363.89–573.2)	5.05	0.0246	ref
Lower than average	17 835	36	201.85 (135.91–267.79)			1.28 (0.77–2.11)	116	650.41 (532.04–768.77)			1.39 (1.04–1.86)
Smoking history^a^											
Never smoke	27 493	44	160.04 (112.75–207.33)	2.55	0.2788	ref	130	472.85 (391.56–554.13)	17.30	0.0002	ref
Prior smoker	1663	4	240.53 (65.54–615.75)			1.55 (0.56–4.31)	13	781.72 (356.77–1206.67)			1.71 (0.96–3.02)
Current smoker	5104	13	254.7 (116.24–393.16)			1.63 (0.88–3.04)	47	920.85 (657.58–1184.11)			2.01 (1.44–2.80)
Drinking history^b^											
Never drink	27 670	49	177.09 (127.50–226.67)	0.32	0.8533	ref	144	520.42 (435.42–605.42)	3.20	0.2017	ref
Prior alcohol user	1234	3	243.11 (50.16–710.70)			1.36 (0.42–4.37)	9	729.34 (333.87–1384.12)			1.39 (0.71–2.73)
Current alcohol user	5313	9	169.4 (77.55–321.48)			0.97 (0.48–1.98)	37	696.41 (472.01–920.8)			1.36 (0.95–1.96)
BMI											
< 18.5	3639	15	412.2 (203.60–620.8)	15.01	0.0005	2.07 (1.12–3.82)	47	1291.56 (922.31–1660.82)	63.49	< 0.0001	1.91 (1.35–2.69)
18.5–23.9	20 884	38	181.96 (124.10–239.81)			ref	130	622.49 (515.48–729.49)			ref
≥ 24	9746	9	92.35 (42.27–175.25)			0.52 (0.25–1.08)	16	164.17 (83.73–244.61)			0.27 (0.16–0.46)
Diabetes											
No	31 867	54	169.45 (124.26–214.65)	3.31	0.0688	ref	177	555.43 (473.61–637.26)	0.49	0.4846	ref
Yes	2402	8	333.06 (143.63–656.12)			1.97 (0.94–4.14)	16	666.11 (339.72–992.51)			1.20 (0.72–2.01)
Close contacts											
No	34 175	62	181.42 (136.26–226.58)		1.0000^d^	ref	190	555.96 (476.91–635.02)		0.0162^d^	ref
Yes	94	0	0 (0–3925.53)			2.89 (0.18–47.03)	3	3191.49 (658.51–9329.79)			5.90 (1.85–18.79)
Previous TB cases											
No	33 663	50	148.53 (107.36–189.70)	110.59	< 0.0001	ref	160	475.3 (401.65–548.95)	262.59	< 0.0001	ref
Yes	606	12	1980.2 (859.80–3100.60)			13.58 (7.20–25.63)	33	5445.54 (3587.57–7303.52)			12.06 (8.22–17.70)
Chronic bronchitis^c^											
No	32 521	58	178.35 (132.45–224.25)		0.7694^d^	ref	175	538.11 (458.39–617.84)	3.94	0.0473	ref
Yes	1647	3	182.15 (37.58–532.48)			0.98 (0.31–3.13)	15	910.75 (449.85–1371.65)			0.59 (0.35–1.00)

*BMI* Body mass index, *CI* Confidence interval, *OR* Odds ratio, *PAF* Population attributable fraction, *TB* Tuberculosis

^a^9 missing

^b^52 missing

^c^101 missing

^d^Fisher’s Exact Test

### Multivariable logistic regression analysis for independent determinants of active PTB

For active PTB, after adjustment in multivariable analysis, sex (M:F) (*OR* = 3.26, 95% *CI*: 2.34–4.55); using age group 65–74 as reference, age group 75–84 (*OR* = 1.59, 95% *CI*: 1.17–2.17), and age group ≥85 (*OR* = 2.05, 95% *CI*: 1.25–3.36); living in rural area (*OR* = 2.65, 95% *CI*: 1.81–3.88); lower family income (*OR*: 1.64, 95% *CI*: 1.17–2.31); using BMI group 18.5–23.9 as reference, BMI group < 18.5 (*OR* = 1.55, 95% *CI*: 1.09–2.22), and BMI group ≥24 (*OR*: 0.33, 95% *CI*: 0.19–0.55); diabetes (*OR* = 1.83, 95% *CI*: 1.08–3.10); close contacts (*OR* = 7.30, 95% *CI*: 2.15–24.82); and previous TB cases (*OR* = 9.23, 95% *CI*: 6.16–13.83), remained strongly associated (Table 4).

**Table 4:** Multivariable logistic regression analysis of active PTB and PAF of high-risk factors among the elderly participants aged 65 or over in China in 2013

Category	Number of participants	Proportion (%)	Adjusted *OR* (95% *CI*)	PAF (%)
Sex				
Female	18 212	53.14	ref	–
Male	16 057	46.86	3.26 (2.34–4.55)	51.43
Age(years)				
65–74	21 685	63.28	ref	–
75–84	10 658	31.10	1.59 (1.17–2.17)	15.51
≥ 85	1926	5.62	2.05 (1.25–3.36)	5.57
Residence				
Urban	12 933	37.74	ref	–
Rural	21 336	62.26	2.65 (1.81–3.88)	50.67
Family annul income per capita				
Higher than average	10 854	31.67	ref	–
Lower than average	23 415	68.33	1.64 (1.17–2.31)	30.42
BMI				
< 18.5	3639	10.62	1.55 (1.09–2.22)	5.52
18.5–23.9	20 884	60.94	ref	–
≥ 24	9746	28.44	0.33 (0.19–0.55)	–
Diabetes				
No	31 867	92.99	ref	–
Yes	2402	7.01	1.83 (1.08–3.10)	5.50
Close contacts				
No	34 175	99.73	ref	–
Yes	94	0.27	7.30 (2.15–24.82)	1.70
Previous TB cases				
No	33 663	98.23	ref	–
Yes	606	1.77	9.23 (6.16–13.83)	12.70

*BMI* Body mass index, *CI* Confidence interval, *OR* Odds ratio, *PAF* Population attributable fraction, *TB* Tuberculosis, *PTB* Pulmonary tuberculosis

PAFs were considered for eight risk factors, as shown in Table 4. Male sex had the highest PAF (51.43%), followed by living in a rural area (50.67%), lower than average family annul income per capita (30.42%), previous TB cases (12.70%), underweight (5.52%), diabetes (5.50%), and close contacts (1.70%). PAFs for age were 15.51% for 75–84 years, and 5.57% for ≥85 years.

## Discussion

This study found that the TB prevalence of elderly people is very high in China, and confirmed that male sex, older age, living in rural areas, underweight, diabetes, close contact of PTB and previously TB history are high risk factors. The risk of TB increased with increasing age and decreasing BMI after adjusting for other factors, and there is a positive dose–response relationship.

Male sex is a strong risk factor for TB disease, supported by both this study and studies from high-income and low-income countries [2, 17–19]. A study from the United States found that, among older adults aged 65 and above, TB rates increased with age [18], which was in accordance with our study. Residence in rural areas and with lower family incomes were risk factors in this study. However, this is not supported by studies from other low-income countries [3, 4, 20, 21]. This may be because in China, the difference between living in urban versus rural areas are more significant than other Southeast Asian and African countries.

Our study showed TB risk decreased with increasing BMI, which is also found in two other studies [22, 23]. TB and diabetes mellitus comorbidity is universal globally, and older age is a risk factor for this comorbidity [24]. Jeon and Murray found the relative risk of diabetes was 3.11 (95% *CI*: 2.27–4.26) [25], higher than this study. A systematic review by Morrison et al. of 41 studies showed that 4.5% of TB patient’s household contacts were diagnosed with active TB [26], and another review by Shah et al. of 25 studies showed the pooled yield was 7.8% (95% *CI*: 5.6–10.0%) for active TB in household contacts of drug-resistant TB [27]. In our study, 3.2% of TB patient’s household contacts were diagnosed with active TB, and the *OR* was 7.30 (95% *CI*: 2.15–24.82). In total, 5.4% (33/193) of previous TB patients had a recurrence, with a high *OR* of 9.23 (95% *CI*: 6.16–13.83) in this study. A retrospective cohort study conducted in Shanghai, China, showed 5.3% (710/13417) of successfully treated cases had a recurrence, a rate of 7.55 (95% *CI*: 7.01–8.13) episodes per 1000 person-years, more than 18 times the rate of TB in the general population [28]. More attention should be paid to patients with a history of previous TB diagnosis.

Heavy alcohol use and alcohol use disorders have a strong association with TB [29, 30], and this study showed the pooled relative risk was 2.94 (95% *CI*: 1.89–4.59), which used an exposure cut-off level set at 40 g of alcohol per day or above, or defined exposure as a clinical diagnosis of an alcohol use disorder [29]. In our study, there was no association between alcohol use and TB, no matter if the alcohol use was prior to or during the study. We defined alcohol use as using 20 g alcohol per week or above, which was much lower than the above-mentioned study. This may have led to the difference. Systematic reviews also showed tobacco smoking and chronic respiratory disease were associated with an increased risk of TB [31, 32]. Our study found the TB prevalence was higher in tobacco smokers and patients with chronic bronchitis, but with no significant statistical difference.

Lönnroth et al. estimated PAFs of selected TB risk factors for 22 high-burden countries, and the PAFs of underweight and diabetes for the total Chinese population were 16.5 and 4.4% respectively [10]. The PAF of underweight in our study was different from that estimation, because of a lower exposure rate and different target population. A study in Zimbabwe had a similar PAF of male sex (40%) and household contacts (10%), but with a high PAF of HIV (33%) [33]. Our study came to the same conclusion as a study in Tanzania [34], that a factor strongly associated with an outcome on an individual level, such as close contact and TB (*OR* = 7.30, PAF = 1.70%), may not have much population impact, if the exposure is not common in the population at large.

Dye and Williams thought that control programs had been less effective than expected in cutting transmission mainly because patients were not diagnosed and cured quickly enough [35]. To find more undiagnosed TB cases, ACF is an effective method. However, ACF can be a costly undertaking, depending on the target population and the diagnostic strategy used [36]. So, the first step to implement ACF is to confirm the target population with high enough prevalence. This study indicated that ACF could be implemented among elderly people aged 65 and above with low BMI (< 18.5), diabetes, close contact history and previous TB history as a priority, because of the higher risks and minor population number. The proportions of low BMI (< 18.5), diabetes, close contact history and previous TB history among elderly people are 10.62, 7.01, 0.27 and 1.77% respectively. Conducting ACF among these high-risk groups of elderly people will be more efficient and cost-effective. For other high-risk factors with a large population number, like male sex or residence in rural areas, it will be not as easy to screen them all. However, high-risk factors combination will be a better choice, which can increase the risk of development of recent active TB and narrow the target population [37].

The strengths of this study were that it was a carefully designed and implemented survey, which used the current TB diagnostic protocols and tests in China for diagnosis. And the results may represent the prevalence and case distribution characteristics of TB among elderly people in China. The study also showed the yield of ACF. The study followed the Strengthening the Reporting of Observational Studies in Epidemiology (STROBE) guidelines [38] and sound ethics principles for the conduct and reporting of this study [39].

The study had a few limitations. Some TB risk factors were collected by self-reporting (such as tobacco use and drinking history) or based on self-reporting and local health documents (such as diabetes), which might not be sufficient to estimate the real distribution among the elderly people. The prevalence of diabetes in this study was 7%, much lower than the 10.9% of the national diabetes prevalence survey in China in 2013 [40]. Meanwhile, our study was unable to detect any association between HIV and TB, as there was only one person who was living with HIV.

## Conclusions

The TB prevalence of elderly people is very high in China, and male sex, older age, living in rural areas, underweight, diabetes, close contact of PTB and previous TB history are high risk factors. The risk of TB increased with increasing age and decreasing BMI after adjusting for other factors, and there is a positive dose–response relationship. ACF could be implemented among elderly people aged 65 and above with underweight, diabetes, close contact history and previous TB history as a priority, which will get significant yields and be cost-effective.

## Additional file

Additional file 1: Multilingual abstracts in the five official working languages of the United Nations. (PDF 242 kb)

## Abbreviations

ACF: Active case finding
AIDS: Acquired immune deficiency syndrome
BMI: Body mass index
*CI*: Confidence interval
CXR: Chest X-ray
HIV: Human immunodeficiency virus
*OR*: Odds ratio
PAF: Population attributable fraction
PTB: Pulmonary tuberculosis
TB: Tuberculosis
