# Supplementary material for: Prevalence and risk factors of active pulmonary tuberculosis among elderly people in China: a population based cross-sectional study
Source: Infect Dis Poverty. 2019 Jan 18;8:7. doi: 10.1186/s40249-019-0515-y (PMC6337869; doi:10.1186/s40249-019-0515-y)
Supplement: Supplementary file 1 — Multilingual abstracts in the five official working languages of the United Nations. (PDF 242 kb) [file 40249_2019_515_MOESM1_ESM.pdf]

Translation of the abstract into the five official working languages of the United Nations

اسباب انتشار وعوامل خطورة الإصابة بالسل الرئوي النشط بين كبار السن في الصين: دراسة شاملة لعدة قطاعات سكانية

Can-You Zhang, Fei Zhao, Yin-Yin Xia, Yan-Ling Yu, Xin Shen, Wei Lu, Xiao-Meng Wang, Jin Xing, Jian-Jun Ye, Jian-Wei Li, Fei-Ying Liu, Jian-Lin Wu, Lin Xu, Hui Zhang, Jun Cheng and Li-Xia Wang

نبذة مختصرة

معلومات أساسيّة: تُعدّ مشكلة الشيخوخة السكانية شاغلًا حيويًا للصحة العامة في الصين الحديثة، ولابد من اتخاذ مزيدًا من الجهود لمكافحة السل وللوصول إلى كبار السن لأعلى الأولويات. في هذه الدراسة، نهدف إلى تحديد اسباب انتشار السل الرئوي وعوامل خطوره الإصابة به بين السكان كبار السن في الصين.

المنهجية: أجريت دراسة مستعرضة متعددة المراحل في عام 2013، واختير 27 قطاع من بين 10 مقطاعات ضمن 10 محافظات صينية. وتم عرض جميع الأشخاص المشاركين أصحاب الفئة العمرية المساوية لـ65 سنة أو أكبر، للفحص على جهاز الأشعة السينية (CXR) واستبيان أعراض المرض. ثلاث عينات جُمعت للفحص البكتريولوجي من خلال المجهر أو المزرعة، كانت نتائجهم إيجابية. تم حساب الانتشار ، "تم إجراء نموذج الانحدار اللوجستي المتعدد لتأكيد عوامل الخطر" ، وتم حساب الجزء المنسوب للسكان (PAF) لكل عامل خطر للإشارة إلى أهمية الصحة العامة.

النتائج: من أصل 388 88 شخصاً مؤهلاً من 27 مجموعة ، أنهى 349 26 مشاركاً الاستبيان والفحص البدني. كان هناك 193 حالة من حالات السل الرئوي النشط ، 62 منها تم تأكيدها جرثوميًا. كان الانتشار التقديري لمرض السل النشط ومرض السل المؤكد البكتريا في تلك الفئة العمرية 65 سنة وما فوقها 563.19 لكل 100000 (مجال الموثوقية 95٪: 483.73 – 642.65) و 180.92 لكل 100000 (95٪ CI: 135.89–225.96) على التوالي. جنس الرجال ، والشيخوخة ، والعيش في المناطق الريفية ، ونقص الوزن ، ومرض السكري ، والاتصال الوثيق بين السل الرئوي (PTB) والتاريخ السابق للسل ، كلها عوامل خطر للإصابة بالسل. ازداد خطر الإصابة بالسل مع زيادة العمر وانخفاض مؤشر كتلة الجسم (BMI) بعد ضبط عوامل أخرى ، وهناك علاقة إيجابية بين الجرعة والاستجابة.

الاستنتاجات: في الصين ، يمكن تنفيذ اكتشاف الحالات النشطة (ACF) بين المسنين الذين تتراوح أعمارهم بين 65 سنة وما فوق مع نقص الوزن ، ومرض السكري ، وتاريخ الاتصال الوثيق ، والتاريخ السل السابق كأولوية ، والتي سوف تحصل على عائدات كبيرة وتكون فعالة من حيث التكلفة.

Translated from English version into Arabic by Mohamed Fouad and Malika, through

TRANSLATORS
WITHOUT BORDERS

中国老年人活动性肺结核的患病率及危险因素：一项基于人群的横断面研究

张灿有，赵飞，夏愔愔，于艳玲，沈鑫，陆伟，王晓萌，邢进，叶建君，李建伟，刘飞鹰，吴建林，许琳，张慧，成君，王黎霞

摘要

引言：人口老龄化是当今中国一个重要的公共卫生关切，应优先针对老年人采取更多的结核病控制措施。本研究拟获得中国老年人中肺结核的患病率和危险因素。

方法：中国于 2013 年开展了一项多阶段整群抽样的横断面调查，选取了 10 个省份 10 个县

区中的 27 个调查点。所有大于等于 65 岁的老年人在签署知情同意后同时接受肺结核可疑症状筛查和胸片检查。有肺结核可疑症状或胸片检查异常者留取 3 份痰标本进行涂片和培养检测。通过计算获得患病率，进行多因素回归分析获得危险因素，通过计算人群归因危险分值分析不同危险因素的公共卫生意义。
结果：27 个调查点中共有 38888 名老年人，其中 34269 名完成了问卷调查和体检。调查共发现 193 例肺结核患者，其中 62 例为细菌学确诊患者。估算 65 岁及以上老年人的活动性肺结核患病率和菌阳肺结核患病率分别为 563.19/10 万 (95%CI:483.73-642.65) 和 180.92 /10 万 (95%CI:135.89-225.96). 男性、高年龄组、农村居民、营养不良、糖尿病、活动性肺结核患者密切接触者和既往结核病患者是肺结核的危险因素。肺结核的发病风险随着年龄的提高和体质指数的降低而增加，存在明确的剂量反应关系。
结论：在中国伴有营养不良、糖尿病、密切接触史、既往结核病患病史的老年人中开展结核病主动发现工作可以得到较高的收益，并且符合成本效益原则。

Translated from English version into Chinese by Can-You Zhang

Prévalence et facteurs de risque de la tuberculose pulmonaire active chez les personnes âgées en Chine : étude transversale de la population chinoise

Can-You Zhang, Fei Zhao, Yin-Yin Xia, Yan-Ling Yu, Xin Shen, Wei Lu, Xiao-Meng Wang, Jin Xing, Jian-Jun Ye, Jian-Wei Li, Fei-Ying Liu, Jian-Lin Wu, Lin Xu, Hui Zhang, Jun Cheng et Li-Xia Wang

Résumé
Contexte : Le vieillissement de la population est un problème critique de santé publique dans la Chine moderne, et la priorité doit être donnée à des efforts supplémentaires de lutte contre la tuberculose (TB) à destination des personnes âgées. Dans cette étude, nous avons voulu déterminer la prévalence et identifier les facteurs de risque de la tuberculose chez les personnes âgées en Chine.
Méthodes : Une enquête transversale par échantillonnage en grappes en plusieurs étapes a été menée en 2013 ; 27 groupes ont été sélectionnés dans 10 circonscriptions de 10 provinces chinoises. Tous les participants consentants âgés de 65 ans ou plus ont subi un dépistage de la tuberculose pulmonaire au moyen d'une radiographie pulmonaire et d'un questionnaire sur les symptômes. Trois échantillons de crachats pour examen bactériologique par microscopie et culture ont été prélevés chez les sujets dont le dépistage a été positif. La prévalence a été calculée, un modèle de régression logistique multiple a été réalisé pour confirmer les facteurs de risque et la fraction attribuable à la population de chaque facteur de risque a été calculée pour indiquer son impact sur la santé publique.
Résultats : Sur 38 888 personnes retenues dans à 27 clusters, 34 269 ont complété le questionnaire et passé l'examen médical. Nous avons recensé 193 cas de tuberculose pulmonaire active, dont 62 ont été confirmés bactériologiquement. La prévalence estimée de la tuberculose pulmonaire active et de la tuberculose bactériologiquement confirmée chez les personnes de 65 ans et plus était de 563,19 cas pour 100 000 personnes (*IC* à 95 % de 483,73 à 642,65) et 180,92 pour 100 000 (*IC* à 95 %de 135,89 à 225,96), respectivement. Le sexe masculin, l'âge avancé, le fait de vivre en milieu rural, l'insuffisance pondérale, le diabète, le contact étroit avec des cas de tuberculose pulmonaire et des antécédents de tuberculose sont tous des facteurs de risque de tuberculose. Le risque de

tuberculose augmentait avec l’âge et la diminution de l’indice de masse corporelle (IMC) après ajustement pour tenir compte d’autres facteurs, et il existe une relation dose-effet positive.
Conclusions : Une recherche de cas actifs pourrait être effectuée en Chine parmi les personnes âgées de 65 ans et plus, en donnant la priorité aux personnes en insuffisance pondérale, diabétiques, ayant eu des contacts étroits avec des patients ou des antécédents de tuberculose afin d’obtenir une bonne efficacité de façon économique.

Translated from English version into French by GABRIELA KOUAHLA and Suzanne Assenat, through

TRANSLATORS
WITHOUT BORDERS

Распространение и факторы риска открытой формы туберкулёза лёгких среди пожилых людей в Китае: поперечное исследование показателей населения

Цань-Ю Чжан, Фэй Чжао, Инь-Инь Ся, Янь-Лин Юй, Синь Шэнь, Вэй Лу, Сяо-Мэн Ван, Цзинь Син, Цзянь-Цзюнь Е, Цзянь-Вэй Ли, Фэй-Ин Лю, Цзянь-Линь У, Линь Сюй, Хуэй Чжан, Цзюнь Чэн и Ли-Ся Ван

Аннотация
Справочная информация: На сегодняшний день, демографическое старение – это серьёзная проблема общественного здравоохранения в Китае, и поэтому необходимо усилить борьбу с туберкулёзом (ТБ), чтобы, в первую очередь, обезопасить пожилых людей. Цель данного исследования – определить, насколько распространён ТБ среди пожилых людей в Китае, а также выявить факторы риска заболевания.
Методы: В 2013 году было проведено групповое поперечное обследование, состоящее из нескольких этапов. С этой целью мы выбрали 27 групп из 10 районов 10 провинций Китая. Всех участников в возрасте от 65 лет и старше обследовали на выявление ТБ с помощью рентгена грудной клетки (РГИ). Кроме того, им предложили пройти симптоматический опросник. У тех, чьи результаты оказались положительными, взяли три образца мокроты для бактериологического исследования с использованием микроскопии. Был вычислен показатель распространение ТБ, для подтверждения факторов риска был применен метод множественной логистической регрессии, и мы рассчитали добавочную долю популяционного риска (ДДПР) для каждого фактора, чтобы подчеркнуть важную роль здравоохранения.
Результаты исследования: Из 38 888 человек (27 обследованных групп) 34 269 участников заполнили опросник и прошли обследование. У 193 участников была обнаружена открытая форма ТБ; из них у 62 участников ТБ был подтвержден бактериологическими анализами. Примерный показатель распространения открытой формы ТБ и бактериологически подтвержденного ТБ у людей от 65 и старше составляет 563,19 на 100000 (95% *ДИ (доверительный интервал)*: 483,73–642,65) и 180,92 на 100000 (95% *ДИ*: 135,89–225,96),

соответственно. Принадлежность к мужскому полу, старение, проживание в сельской местности, дефицит массы тела, диабет, тесный контакт с больными ЛТБ и наличие ранней истории болезни – всё это факторы риска ТБ. Риск заражения, помимо прочих показателей, увеличился из-за старения и уменьшения индекса массы тела (ИМТ). К тому же, установлена позитивная зависимость «доза-эффект».
Выводы: В Китае выявление заболевания на ранних стадиях следует осуществить, в первую очередь, среди пожилых людей от 65 лет и старше с дефицитом массы тела, диабетом, находившихся в тесном контакте с больными или тех, кто ранее перенёс ТБ. Такой подход является экономически выгодным и даст существенные результаты.

Translated from English version into Russian by Lamiya Mehtieva and Natalia Potashnik, through

TRANSLATORS WITHOUT BORDERS

La prevalencia y los factores de riesgo de la tuberculosis pulmonar activa entre las personas de edad avanzada en China: un estudio transversal basado en la población.

Can-You Zhang, Fei Zhao, Yin-Yin Xia, Yan-Ling Yu, Xin Shen, Wei Lu, Xiao-Meng Wang, Jin Xing, Jian-Jun Ye, Jian-Wei Li, Fei-Ying Liu, Jian-Lin Wu, Lin Xu, Hui Zhang, Jun Cheng y Li-Xia Wang

Resumen
Introducción: El problema del envejecimiento de la población es una principal preocupación de salud pública en la China actual y se necesitan más esfuerzos de control de la tuberculosis (TB) que lleguen a las personas ancianas con la máxima prioridad. En este estudio, nos proponemos determinar la prevalencia e identificar los factores de riesgo de la TB entre las personas ancianas en China.
Metodología: En 2013 se realizó una encuesta transversal usando un muestreo por conglomerados en etapas múltiples y se seleccionaron 27 grupos de 10 condados de 10 provincias chinas. Se examinó de TB pulmonar a todos los participantes de 65 años de edad o mayores que dieron su consentimiento mediante una radiografía de tórax (RXT) y un cuestionario sobre los síntomas. Se recolectaron tres muestras de esputo de aquellos participantes cuyo cribado dio positivo para hacer un examen bacteriológico mediante microscopía y cultivo. Se calculó la prevalencia, se llevó a cabo un modelo de regresión logística múltiple para confirmar los factores de riesgoy se calculó la fracción atribuible poblacional (FAP) de cada factor de riesgo para indicar su importancia en la salud pública.
Resultados: De las 38.888 personas eligibles en los 27 grupos, 34.269 participantes terminaron tanto el cuestionario como la exploración física. Se hallaron 193 casos de TB pulmonar activa, de los cuales 62 fueron casos confirmados bacteriológicamente. La prevalencia estimada de TB pulmonar activa y los casos de TB confirmados bacteriológicamente entre los participantes de 65 años de edad o mayores fueron de 563,19 por 100 000 (IC del 95%: 483,73–642,65) y de 180,92

por 100 000 (IC del 95%: 135,89–225,96) respectivamente. Las personas con factores de riesgo de contraer la TB son aquellas de sexo masculino, ancianas, que viven en zonas rurales, con insuficiencia ponderal, diabetes, en contacto próximo a la TB pulmonar (TBP) y que tienen antecedentes de TB. El riesgo de la TB aumentó con el incremento de la edad y la disminución del índice de masa corporal (IMC) tras el ajuste por otros factores, y existe una relación positiva dosis-respuesta.

Conclusiones: En China, se podría implementar de forma prioritaria una búsqueda activa de casos (ACF, por sus siglas en inglés) entre personas de 65 años y mayores con insuficiencia ponderal, diabetes, antecedentes de contacto próximo y de TB, con lo que se conseguirán mejoras significativas y rentables.

Translated from English version into Spanish by Guillermo Pinilla Gallego and Carolina15, through

TRANSLATORS
WITHOUT BORDERS
